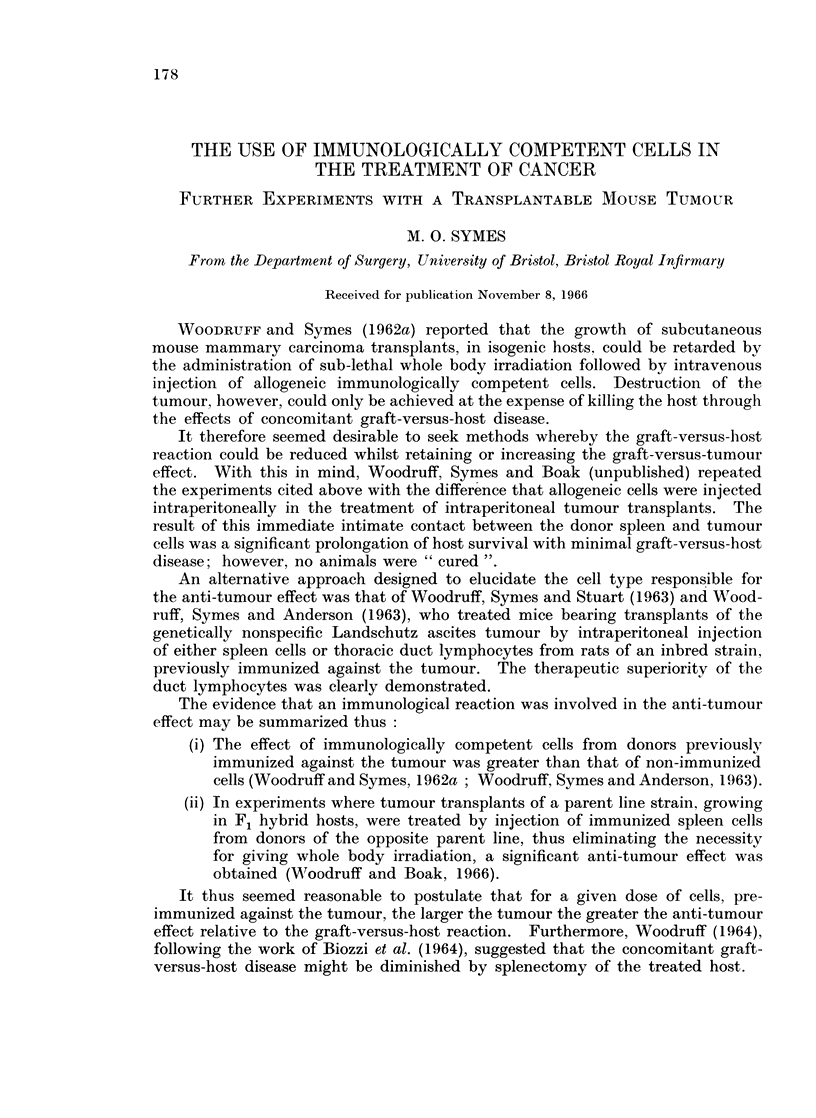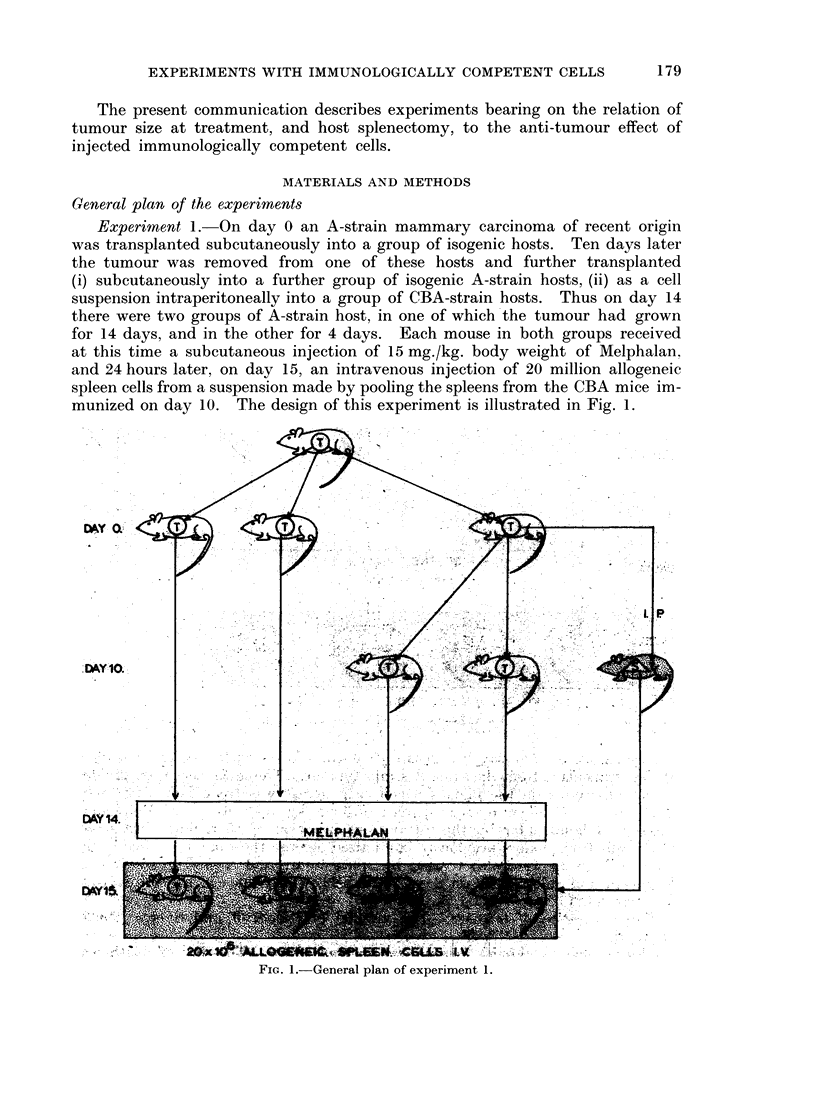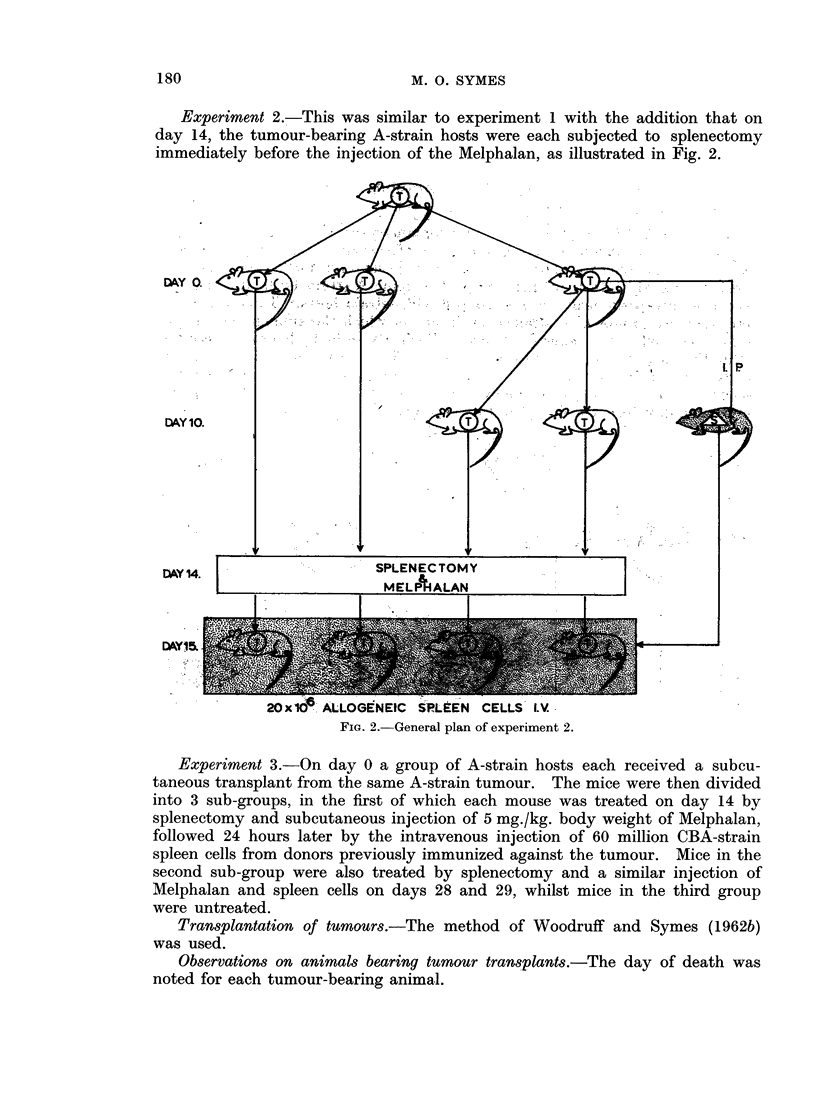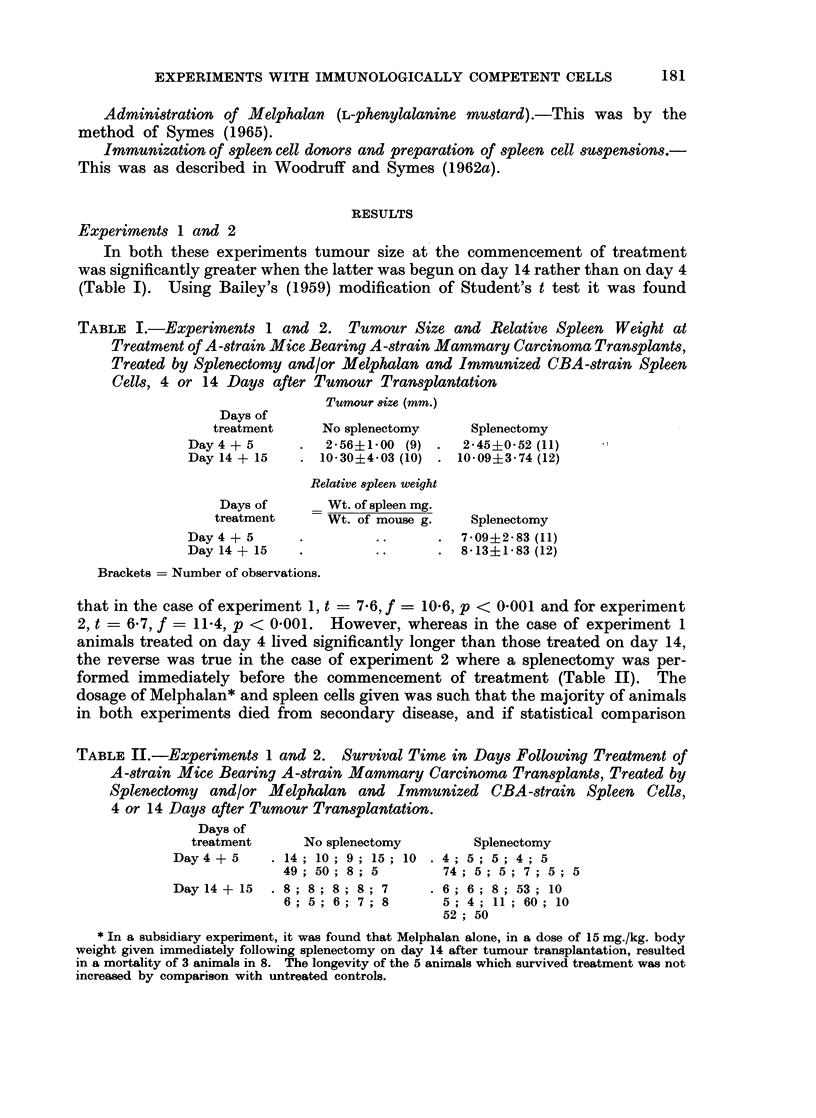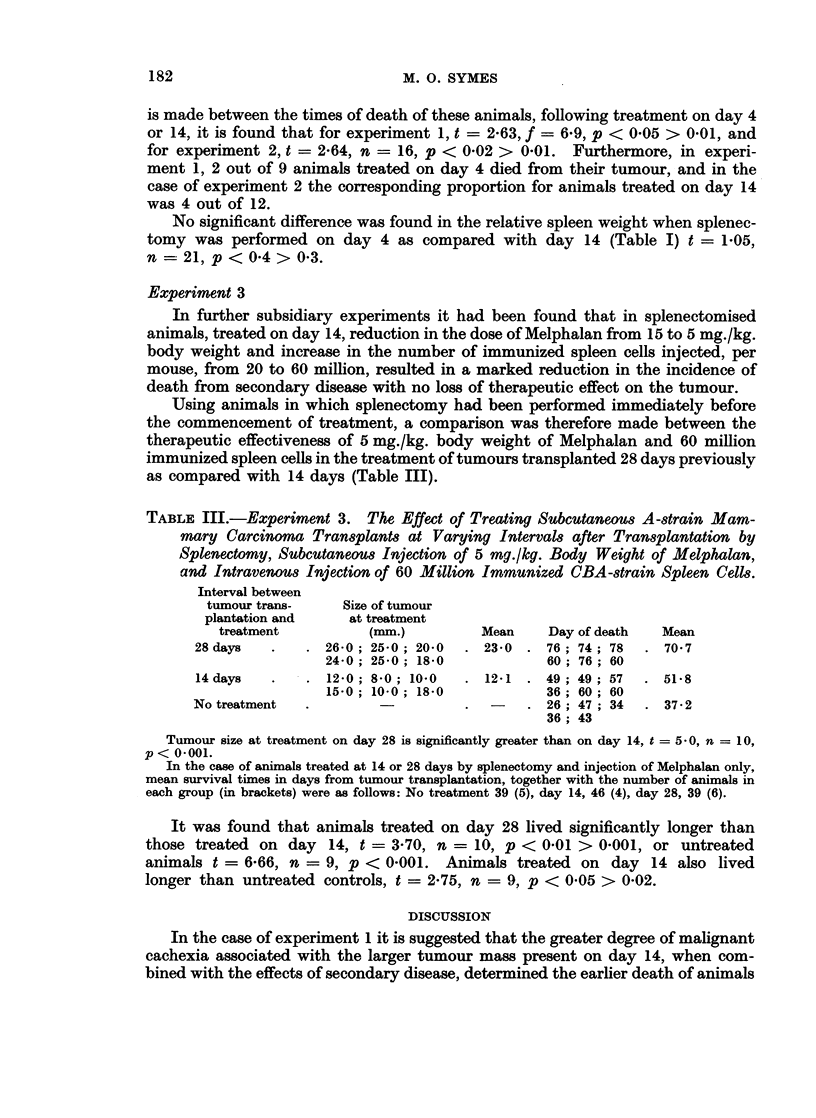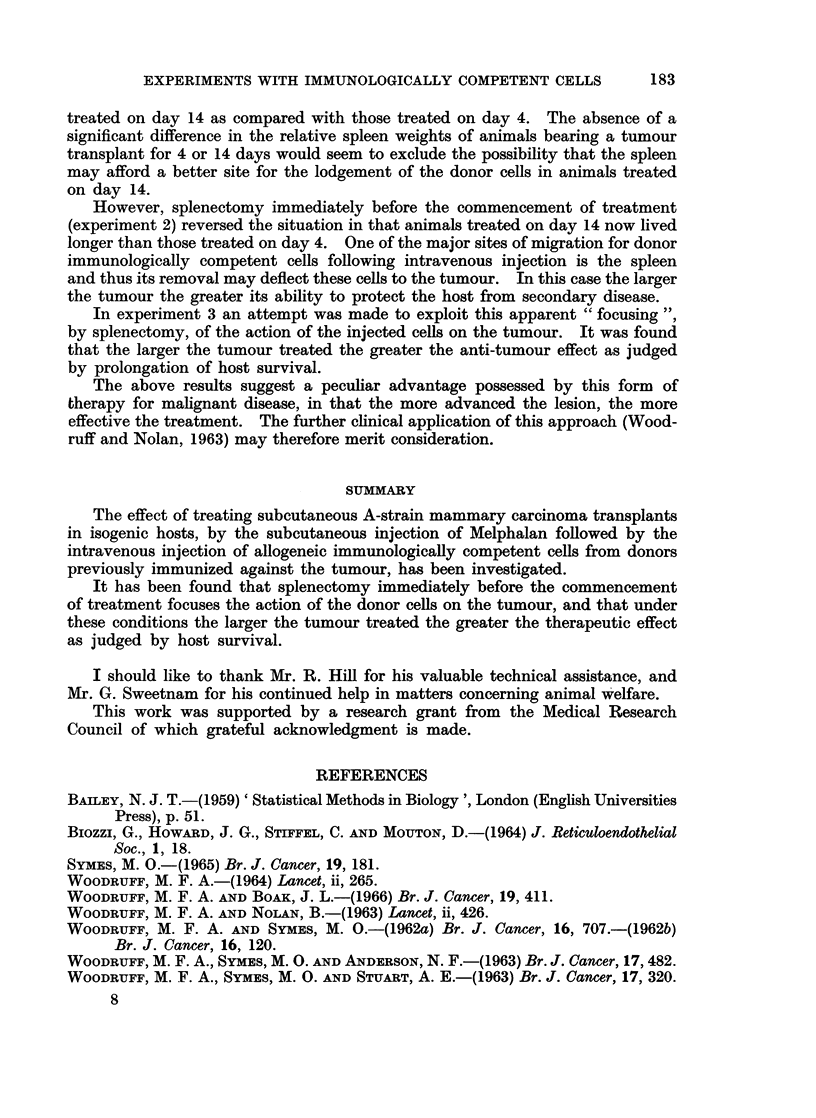# The use of immunologically competent cells in the treatment of cancer. Further experiments with a transplantable mouse tumour.

**DOI:** 10.1038/bjc.1967.18

**Published:** 1967-03

**Authors:** M. O. Symes


### THE USE OF IMMUNOLOGICALLY COMPETENT CELLS IN THE TREATMENT OF CANCER

### FURTHER EXPERIMENTS WITH A TRANSPLANTABLE MOUSE TUMOUR

M. O. SYMES

*From the Department of Surgery, University of Bristol, Bristol Royal Infirmary*

Received for publication November 8, 1966

WOODRUFF and Symes (1962*a*) reported that the growth of subcutaneous mouse mammary carcinoma transplants, in isogenic hosts, could be retarded by the administration of sub-lethal whole body irradiation followed by intravenous injection of allogeneic immunologically competent cells. Destruction of the tumour, however, could only be achieved at the expense of killing the host through the effects of concomitant graft-versus-host disease.

It therefore seemed desirable to seek methods whereby the graft-versus-host reaction could be reduced whilst retaining or increasing the graft-versus-tumour effect. With this in mind, Woodruff, Symes and Boak (unpublished) repeated the experiments cited above with the difference that allogeneic cells were injected intraperitoneally in the treatment of intraperitoneal tumour transplants. The result of this immediate intimate contact between the donor spleen and tumour cells was a significant prolongation of host survival with minimal graft-versus-host disease; however, no animals were " cured ".

An alternative approach designed to elucidate the cell type responsible for the anti-tumour effect was that of Woodruff, Symes and Stuart (1963) and Woodruff, Symes and Anderson (1963), who treated mice bearing transplants of the genetically nonspecific Landschutz ascites tumour by intraperitoneal injection of either spleen cells or thoracic duct lymphocytes from rats of an inbred strain, previously immunized against the tumour. The therapeutic superiority of the duct lymphocytes was clearly demonstrated.

The evidence that an immunological reaction was involved in the anti-tumour effect may be summarized thus :

(i) The effect of immunologically competent cells from donors previously immunized against the tumour was greater than that of non-immunized cells (Woodruff and Symes, 1962*a* ; Woodruff, Symes and Anderson, 1963).

(ii) In experiments where tumour transplants of a parent line strain, growing in $F_1$ hybrid hosts, were treated by injection of immunized spleen cells from donors of the opposite parent line, thus eliminating the necessity for giving whole body irradiation, a significant anti-tumour effect was obtained (Woodruff and Boak, 1966).

It thus seemed reasonable to postulate that for a given dose of cells, pre-immunized against the tumour, the larger the tumour the greater the anti-tumour effect relative to the graft-versus-host reaction. Furthermore, Woodruff (1964), following the work of Biozzi *et al.* (1964), suggested that the concomitant graft-versus-host disease might be diminished by splenectomy of the treated host.

The present communication describes experiments bearing on the relation of tumour size at treatment, and host splenectomy, to the anti-tumour effect of injected immunologically competent cells.

### MATERIALS AND METHODS

### *General plan of the experiments*

*Experiment* 1.—On day 0 an A-strain mammary carcinoma of recent origin was transplanted subcutaneously into a group of isogenic hosts. Ten days later the tumour was removed from one of these hosts and further transplanted (i) subcutaneously into a further group of isogenic A-strain hosts, (ii) as a cell suspension intraperitoneally into a group of CBA-strain hosts. Thus on day 14 there were two groups of A-strain host, in one of which the tumour had grown for 14 days, and in the other for 4 days. Each mouse in both groups received at this time a subcutaneous injection of 15 mg./kg. body weight of Melphalan, and 24 hours later, on day 15, an intravenous injection of 20 million allogeneic spleen cells from a suspension made by pooling the spleens from the CBA mice immunized on day 10. The design of this experiment is illustrated in Fig. 1.

FIG. 1.—General plan of experiment 1.

*Experiment* 2.—This was similar to experiment 1 with the addition that on day 14, the tumour-bearing A-strain hosts were each subjected to splenectomy immediately before the injection of the Melphalan, as illustrated in Fig. 2.

FIG. 2.—General plan of experiment 2.

*Experiment* 3.—On day 0 a group of A-strain hosts each received a subcutaneous transplant from the same A-strain tumour. The mice were then divided into 3 sub-groups, in the first of which each mouse was treated on day 14 by splenectomy and subcutaneous injection of 5 mg./kg. body weight of Melphalan, followed 24 hours later by the intravenous injection of 60 million CBA-strain spleen cells from donors previously immunized against the tumour. Mice in the second sub-group were also treated by splenectomy and a similar injection of Melphalan and spleen cells on days 28 and 29, whilst mice in the third group were untreated.

*Transplantation of tumours*.—The method of Woodruff and Symes (1962*b*) was used.

*Observations on animals bearing tumour transplants*.—The day of death was noted for each tumour-bearing animal.

*Administration of Melphalan* (L-*phenylalanine mustard*).—This was by the method of Symes (1965).

*Immunization of spleen cell donors and preparation of spleen cell suspensions.*—This was as described in Woodruff and Symes (1962*a*).

### RESULTS

*Experiments* 1 *and* 2

In both these experiments tumour size at the commencement of treatment was significantly greater when the latter was begun on day 14 rather than on day 4 (Table I). Using Bailey's (1959) modification of Student's $t$ test it was found

TABLE I.—*Experiments* 1 *and* 2. *Tumour Size and Relative Spleen Weight at Treatment of A-strain Mice Bearing A-strain Mammary Carcinoma Transplants, Treated by Splenectomy and/or Melphalan and Immunized CBA-strain Spleen Cells,* 4 *or* 14 *Days after Tumour Transplantation*

| Days of treatment | Tumour size (mm.) No splenectomy | Tumour size (mm.) Splenectomy |
|---|---|---|
| Day 4 + 5 | 2·56±1·00 (9) | 2·45±0·52 (11) |
| Day 14 + 15 | 10·30±4·03 (10) | 10·09±3·74 (12) |

| Days of treatment | Relative spleen weight $= \frac{\text{Wt. of spleen mg.}}{\text{Wt. of mouse g.}}$ | Splenectomy |
|---|---|---|
| Day 4 + 5 | .. | 7·09±2·83 (11) |
| Day 14 + 15 | .. | 8·13±1·83 (12) |

Brackets = Number of observations.

that in the case of experiment 1, $t = 7{\cdot}6, f = 10{\cdot}6, p < 0{\cdot}001$ and for experiment 2, $t = 6{\cdot}7, f = 11{\cdot}4, p < 0{\cdot}001$. However, whereas in the case of experiment 1 animals treated on day 4 lived significantly longer than those treated on day 14, the reverse was true in the case of experiment 2 where a splenectomy was performed immediately before the commencement of treatment (Table II). The dosage of Melphalan* and spleen cells given was such that the majority of animals in both experiments died from secondary disease, and if statistical comparison

TABLE II.—*Experiments* 1 *and* 2. *Survival Time in Days Following Treatment of A-strain Mice Bearing A-strain Mammary Carcinoma Transplants, Treated by Splenectomy and/or Melphalan and Immunized CBA-strain Spleen Cells,* 4 *or* 14 *Days after Tumour Transplantation.*

| Days of treatment | No splenectomy | Splenectomy |
|---|---|---|
| Day 4 + 5 | 14 ; 10 ; 9 ; 15 ; 10<br>49 ; 50 ; 8 ; 5 | 4 ; 5 ; 5 ; 4 ; 5<br>74 ; 5 ; 5 ; 7 ; 5 ; 5 |
| Day 14 + 15 | 8 ; 8 ; 8 ; 8 ; 7<br>6 ; 5 ; 6 ; 7 ; 8 | 6 ; 6 ; 8 ; 53 ; 10<br>5 ; 4 ; 11 ; 60 ; 10<br>52 ; 50 |

* In a subsidiary experiment, it was found that Melphalan alone, in a dose of 15 mg./kg. body weight given immediately following splenectomy on day 14 after tumour transplantation, resulted in a mortality of 3 animals in 8. The longevity of the 5 animals which survived treatment was not increased by comparison with untreated controls.

is made between the times of death of these animals, following treatment on day 4 or 14, it is found that for experiment 1, $t = 2{\cdot}63$, $f = 6{\cdot}9$, $p < 0{\cdot}05 > 0{\cdot}01$, and for experiment 2, $t = 2{\cdot}64$, $n = 16$, $p < 0{\cdot}02 > 0{\cdot}01$. Furthermore, in experiment 1, 2 out of 9 animals treated on day 4 died from their tumour, and in the case of experiment 2 the corresponding proportion for animals treated on day 14 was 4 out of 12.

No significant difference was found in the relative spleen weight when splenectomy was performed on day 4 as compared with day 14 (Table I) $t = 1{\cdot}05$, $n = 21$, $p < 0{\cdot}4 > 0{\cdot}3$.

*Experiment* 3

In further subsidiary experiments it had been found that in splenectomised animals, treated on day 14, reduction in the dose of Melphalan from 15 to 5 mg./kg. body weight and increase in the number of immunized spleen cells injected, per mouse, from 20 to 60 million, resulted in a marked reduction in the incidence of death from secondary disease with no loss of therapeutic effect on the tumour.

Using animals in which splenectomy had been performed immediately before the commencement of treatment, a comparison was therefore made between the therapeutic effectiveness of 5 mg./kg. body weight of Melphalan and 60 million immunized spleen cells in the treatment of tumours transplanted 28 days previously as compared with 14 days (Table III).

TABLE III.—*Experiment* 3. *The Effect of Treating Subcutaneous A-strain Mammary Carcinoma Transplants at Varying Intervals after Transplantation by Splenectomy, Subcutaneous Injection of* 5 *mg./kg. Body Weight of Melphalan, and Intravenous Injection of* 60 *Million Immunized CBA-strain Spleen Cells.*

| Interval between tumour transplantation and treatment | Size of tumour at treatment (mm.) | Mean | Day of death | Mean |
|---|---|---|---|---|
| 28 days | 26·0 ; 25·0 ; 20·0<br>24·0 ; 25·0 ; 18·0 | 23·0 | 76 ; 74 ; 78<br>60 ; 76 ; 60 | 70·7 |
| 14 days | 12·0 ; 8·0 ; 10·0<br>15·0 ; 10·0 ; 18·0 | 12·1 | 49 ; 49 ; 57<br>36 ; 60 ; 60 | 51·8 |
| No treatment | — | — | 26 ; 47 ; 34<br>36 ; 43 | 37·2 |

Tumour size at treatment on day 28 is significantly greater than on day 14, $t = 5{\cdot}0$, $n = 10$, $p < 0{\cdot}001$.

In the case of animals treated at 14 or 28 days by splenectomy and injection of Melphalan only, mean survival times in days from tumour transplantation, together with the number of animals in each group (in brackets) were as follows: No treatment 39 (5), day 14, 46 (4), day 28, 39 (6).

It was found that animals treated on day 28 lived significantly longer than those treated on day 14, $t = 3{\cdot}70$, $n = 10$, $p < 0{\cdot}01 > 0{\cdot}001$, or untreated animals $t = 6{\cdot}66$, $n = 9$, $p < 0{\cdot}001$. Animals treated on day 14 also lived longer than untreated controls, $t = 2{\cdot}75$, $n = 9$, $p < 0{\cdot}05 > 0{\cdot}02$.

### DISCUSSION

In the case of experiment 1 it is suggested that the greater degree of malignant cachexia associated with the larger tumour mass present on day 14, when combined with the effects of secondary disease, determined the earlier death of animals treated on day 14 as compared with those treated on day 4. The absence of a significant difference in the relative spleen weights of animals bearing a tumour transplant for 4 or 14 days would seem to exclude the possibility that the spleen may afford a better site for the lodgement of the donor cells in animals treated on day 14.

However, splenectomy immediately before the commencement of treatment (experiment 2) reversed the situation in that animals treated on day 14 now lived longer than those treated on day 4. One of the major sites of migration for donor immunologically competent cells following intravenous injection is the spleen and thus its removal may deflect these cells to the tumour. In this case the larger the tumour the greater its ability to protect the host from secondary disease.

In experiment 3 an attempt was made to exploit this apparent " focusing ", by splenectomy, of the action of the injected cells on the tumour. It was found that the larger the tumour treated the greater the anti-tumour effect as judged by prolongation of host survival.

The above results suggest a peculiar advantage possessed by this form of therapy for malignant disease, in that the more advanced the lesion, the more effective the treatment. The further clinical application of this approach (Woodruff and Nolan, 1963) may therefore merit consideration.

### SUMMARY

The effect of treating subcutaneous A-strain mammary carcinoma transplants in isogenic hosts, by the subcutaneous injection of Melphalan followed by the intravenous injection of allogeneic immunologically competent cells from donors previously immunized against the tumour, has been investigated.

It has been found that splenectomy immediately before the commencement of treatment focuses the action of the donor cells on the tumour, and that under these conditions the larger the tumour treated the greater the therapeutic effect as judged by host survival.

I should like to thank Mr. R. Hill for his valuable technical assistance, and Mr. G. Sweetnam for his continued help in matters concerning animal welfare.

This work was supported by a research grant from the Medical Research Council of which grateful acknowledgment is made.

### REFERENCES

BAILEY, N. J. T.—(1959) ' Statistical Methods in Biology ', London (English Universities Press), p. 51.
BIOZZI, G., HOWARD, J. G., STIFFEL, C. AND MOUTON, D.—(1964) *J. Reticuloendothelial Soc.*, **1**, 18.
SYMES, M. O.—(1965) *Br. J. Cancer*, **19**, 181.
WOODRUFF, M. F. A.—(1964) *Lancet*, ii, 265.
WOODRUFF, M. F. A. AND BOAK, J. L.—(1966) *Br. J. Cancer*, **19**, 411.
WOODRUFF, M. F. A. AND NOLAN, B.—(1963) *Lancet*, ii, 426.
WOODRUFF, M. F. A. AND SYMES, M. O.—(1962*a*) *Br. J. Cancer*, **16**, 707.—(1962*b*) *Br. J. Cancer*, **16**, 120.
WOODRUFF, M. F. A., SYMES, M. O. AND ANDERSON, N. F.—(1963) *Br. J. Cancer*, **17**, 482.
WOODRUFF, M. F. A., SYMES, M. O. AND STUART, A. E.—(1963) *Br. J. Cancer*, **17**, 320.